# Statistically Representative Metrology of Nanoparticles via Unsupervised Machine Learning of TEM Images

**DOI:** 10.3390/nano11102706

**Published:** 2021-10-14

**Authors:** Haotian Wen, José María Luna-Romera, José C. Riquelme, Christian Dwyer, Shery L. Y. Chang

**Affiliations:** 1 School of Materials Science and Engineering, University of New South Wales, Sydney, NSW 2052, Australia; 2 Software and Computing Systems, Universidad de Sevilla, 41004 Seville, Spain; jmluna@us.es (J.M.L.-R.); riquelme@us.es (J.C.R.); 3 Electron Imaging and Spectroscopy Tools, Sydney, NSW 2219, Australia; dwyer@eistools.com; 4 Mark Wainwright Analytical Centre, Electron Microscope Unit, University of New South Wales, Sydney, NSW 2052, Australia

**Keywords:** nanoparticles, image analysis, machine learning

## Abstract

The morphology of nanoparticles governs their properties for a range of important applications. Thus, the ability to statistically correlate this key particle performance parameter is paramount in achieving accurate control of nanoparticle properties. Among several effective techniques for morphological characterization of nanoparticles, transmission electron microscopy (TEM) can provide a direct, accurate characterization of the details of nanoparticle structures and morphology at atomic resolution. However, manually analyzing a large number of TEM images is laborious. In this work, we demonstrate an efficient, robust and highly automated unsupervised machine learning method for the metrology of nanoparticle systems based on TEM images. Our method not only can achieve statistically significant analysis, but it is also robust against variable image quality, imaging modalities, and particle dispersions. The ability to efficiently gain statistically significant particle metrology is critical in advancing precise particle synthesis and accurate property control.

## 1. Introduction

Nanoparticles are widely used in applications such as bio-medicine [1,2,3,4], sensors [5,6], nanoscale thermal metrology [7], energy [8], environmental protection [9], optics [10,11,12,13] and electronics [14,15]. The size, shape, and dispersion of the particles play an important role in their properties. Examples include the enhancement of the catalytic activities of nanoparticles by controlling their shape [16,17]; the particle size- and shape-dependent drug molecule absorption in drug delivery [18]; the strong size/shape correlation with their optical properties [19,20]; and control of the size and dispersion of nanoparticles to optimize their photoacoustic effect in the field of bioimaging [21,22,23]. However, current studies generally either lack the accurate particle shape analysis or do not provide a statistically representative number of particles in their measurements. This is particularly problematic as many-nanoparticle syntheses can have large fluctuations within the same batch or across batches [24,25,26]. Therefore, there is an urgent need for an accurate and statistically significant analysis of the size and shape distribution of nanoparticles to aid the control in nanoparticle synthesis, thereby optimizing their desired properties.

Transmission electron microscopy (TEM) offers high spatial resolution imaging and can provide structural information of nanoparticles down to the atomic level [27,28,29]. The technique uses a beam of medium-energy (∼80–300 keV) electrons to image a thin sample. It is well suited for an accurate analysis of nanoparticle shapes and sizes [30]. However, manually analyzing and classifying the shape of particles in TEM images is laborious and challenging, especially when a statistically meaningful number of particles is needed. Some existing image analysis software [31,32] can partially solve this problem by carrying out particle size distribution (PSD) analysis of nanoparticles. Still, nearly all of the existing software lacks the ability to effectively analyze and classify the shape of the particles.

Machine learning (ML) methods have been shown to accomplish efficient classification of large data sets in many fields, such as text mining and biology [33,34]. Currently, deep learning methods such as Convolutional Neural Network (CNN) algorithms [35,36] and Artificial Neural Network (ANN) have been used for particle shape classification [37,38]. Although this approach achieves good results for specific particle shapes, it is dependent on *a priori* knowledge of the latter to generate training datasets. In order to achieve generalization in data analysis, an unsupervised ML algorithm, which does not require prior input and data training, is more desirable. Recently published studies are showing that unsupervised ML methods can efficiently analyze the size and shape information of the nanoparticles; however, they are limited to nanoparticles with well distinguished shapes with strong image contrast (such as gold nano-rods) and good dispersions (isolated particles) [39,40]. In reality, it is difficult to guarantee a clear contrast and a homogeneous background in EM images due to a wide range of nanoparticle compositions, dispersions and imaging conditions.

Here, we demonstrate a robust and efficient unsupervised ML method for nanoparticle shape classification and morphological distribution analysis. The method first uses computer vision algorithms to pre-process images and obtain particle morphology information, followed by a hierarchical-clustering based unsupervised ML algorithm to classify particle shapes and ultimately output statistical particle morphology analysis results. This method is highly automated and it is applicable to convex nanoparticle shapes (this limitation will become clear in Section 2.4), dispersion density and particle composition (which determines the image contrast). Our method is demonstrated using three carefully chosen examples that cover nanoparticles with different shapes, particle dispersions, and TEM imaging modes. By using our presented method, we can overcome the shortcomings of the conventional manual TEM data analysis methods, which analyze an insufficient number of particles, giving poor statistics. Moreover, this method also fills a gap in the particle shape classification in the common large-number particle analysis software.

## 2. Methodology

**Figure 1:**
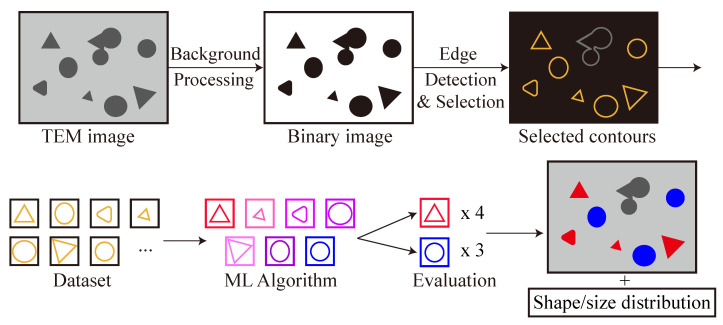
Schematic workflow of the methodology. The upper panel shows the imaging pre-processing steps and the lower panel illustrates the particle shape classification and evaluation via the ML algorithm.

The schematic diagram of our ML method workflow is shown in Figure 1. The method is divided into two parts: The first part is pre-processing, which converts the raw image data into nanoparticle contour datasets that are then applied to the ML algorithm. The second part is the application of the ML algorithm to classify the shapes and the evaluation of the ML output to obtain the relevant information, in this case, the shape distribution.

### 2.1. Sample Preparation and TEM Imaging

The TEM samples of nanoparticles were prepared by drop-casting nanoparticle dispersions onto the holey carbon film coated Cu TEM grids.

The nanoparticle TEM samples were imaged using JEOL F200 (Akishima, Tokyo, Japan), operated at 200 kV, using either the bright-field TEM (BF-TEM) mode (where the objective aperture was placed around the transmitted beam to form an image) or the annular-dark field scanning-TEM (ADF-STEM) mode (where a small probe formed by STEM mode was raster through the specimen and the scattered electrons were collected by the ADF detector to form an image). Note that these modalities in the TEM generate images of the sample as seen in projection, and hence the shapes of nanoparticles extracted using the steps described below are two-dimensional projections.

### 2.2. Pre-Processing: Background Removal

The first step of pre-processing is to separate the particles from the background in the image. One common way to achieve this is to take advantage of the difference in brightness between the particles and the background in the TEM image by setting a global brightness threshold to filter out the background in the image [41]. However, one challenge in relying on this approach to obtain reliable background-removed images is the inhomogeneity of the background. Such inhomogeneity can arise from variations in the thickness, or surface unevenness of the sample background matrix, or from non-uniform electron microscope illumination. The inhomogeneous background can lead to the regional background being over- and under-filtered in their removal process when a global filtering threshold is applied. As a result, the process either does not properly isolate the particles or it introduces artifacts, which cause a misrepresentation of the shapes. Such inadequate background removal can interfere with the accuracy of particle shape information.

**Figure 2:**
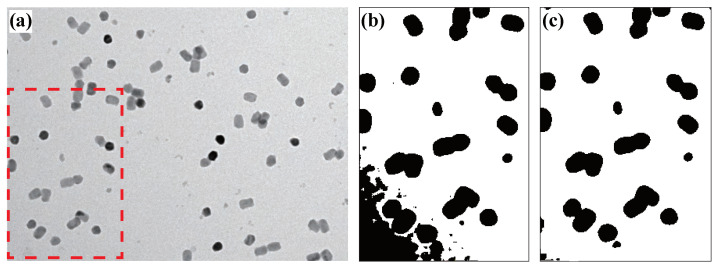
Background pre-processing and removal. (**a**) Raw TEM image with uneven background. (**b**) After global thresholding. The area shown here is highlighted in red dashed line in (**a**). (**c**) After dynamic thresholding of the same area in (**b**).

To overcome this, we adopt a more sophisticated approach of dynamic thresholding. This method measures the local background intensities within optimal sub-regions in the TEM image, then adjusts the background in different small sub-regions of the image to be consistent with the optimal before the final background removal [42]. As an example shown in Figure 2a, due to the uneven background intensity in the image, with a brighter background in the upper right region and a darker background in the lower-left region, the darker region indicated in red dashed lines is under-filtered when a global threshold is applied (Figure 2b).

After using the dynamic thresholding method, the particles that are buried by the under-filtered background are now clearly distinguished (Figure 2c). We will show that this method is effective and robust in Section 3.

### 2.3. Pre-Processing: Particle Edge Identification

After removing the background and converting the images into binary form, the particle’s edges (shapes) are identified by applying the Canny Edge Detection algorithm [43]. This algorithm is based on the first-order derivative of the noise-reduced, smoothed image in the X, Y directions, and the edges of the particles are located at the pixels with maximum values of the first-order derivative along the gradient directions. This is a widely used method due to its simplicity and robustness [44,45].

### 2.4. Pre-Processing: Overlapping Particles Filtering

One limitation of the Canny Edge method is that it cannot correctly identify the particle edges when they are aggregated or overlapped.

Numerous methods, such as watershed segmentation [39], and dilate and flood filling [46], have been developed to identify individual particles from aggregates. These methods work well when the particles are just “touching” but they fail when particles overlap each other. Recently, deep learning methods have been shown to achieve accurate identification and segmentation of highly overlapped particles through data training [47]. However, again, these deep learning methods are not generally applicable without *a priori* knowledge.

Here we use a straight-forward convexity filtering method to simply filter out the aggregated/overlapping particles. This approach assumes that the nanoparticles that are convex and aggregated tend to have concave shapes. By identifying the convexity of the particle contours, aggregates can be detected [48] and then removed from the dataset. This approach ensures meaningful classification and, at the same time, can still retain statistically significant numbers of nanoparticles being analyzed by using mode images. As the convexity filtering places the assumption that the isolated particles are convex shaped, this simple filtering method therefore sets a limitation to our method. Considering concave shaped nanoparticles are relatively uncommon [49], this convexity filtering method can still be applied to most types of nanoparticles. While the limitation of the method is set by the aggregate filtering here, the particle shape classification method (see next section) is not limited to convex shaped particles.

### 2.5. Classification

Having converted the TEM image data into the particle contour dataset, we then apply an unsupervised ML classification algorithm for the particle shape classification. Unsupervised ML algorithms, unlike *supervised* ML methods, do not require prior classified information for data training, thus allowing our method to be directly applied to a wide range of nanoparticles. More discussions regarding the differences between the supervised and unsupervised ML methods can be found in References [50,51].

In the classification process, we firstly parameterized the shape of each particle contour using Hu moments, which are a set of numbers that depend only on the particle shape and are independent of the particle size, position and orientation [52]. Then we applied the hierarchical agglomerative clustering method with the average linkage [53] to classify the parameterized particle shapes. Briefly speaking, a hierarchical clustering algorithm groups together data points (in our case, the particle contours) with similarities between them. This is achieved by using a measurement metric (in our case, the "distance" between the pair of data points) and a linkage criteria, which specifies the similarity of data sets as a function of pairwise distances of observations in the sets (more detail can be found in the Supplementary Information) [54]. This clustering method builds a hierarchy of clusters and therefore does not require a pre-determined optimum numbers of clusters. Such method has been previously applied in other fields [55,56,57]. Such method is different from the commonly used k-means method [58] where users need to decide the number of clusters in advance. Thus, the hierarchical clustering method allows full automation. Finally, the optimum numbers of clusters are determined automatically by applying internal Cluster Validity Indexes (CVIs). The three internal CVIs we choose are Silhouette [59], Davies–Bouldin [60], and Calinski–Harabaz [61], and the optimum numbers of clusters are the common local extrema of each CVI. A detailed description of the internal CVIs can be found in the Supplementary Information .

We emphasize here that our method is an unsupervised machine learning algorithm (as distinct from, e.g., “deep learning” methods), which does not require initial simulations and is therefore generally applicable to any nanoparticle samples without the need of “training data.” In addition, with some initial setup, the method can automatically analyze sets of TEM images containing hundreds of nanoparticles within a few seconds using a personal laptop, which demonstrates its efficiency.

## 3. Results and Discussions

Here, we demonstrate the effectiveness of our method using three examples, which cover different particle shapes, dispersion, and TEM imaging modalities.

### 3.1. Well Dispersed, Multiple Shape Upconversion Nanoparticles

Upconversion nanoparticles (UCNPs) are a unique class of optical nanomaterials that can up-convert two or more low energy photons into one higher energy photon [62]. They have been used in a broad range of applications such as bio-imaging [63], photodynamic theranostics [64,65], and anti-counterfeiting [66]. Lanthanide ion (Ln) doped UCNPs have been demonstrated as one of the most efficient upconversion fluorescent nanoparticles due to their low phonon vibration energy [67,68]. The size and shape of UCNPs play an important role in modifying the optical and electromagnetic properties [69].

**Figure 3:**
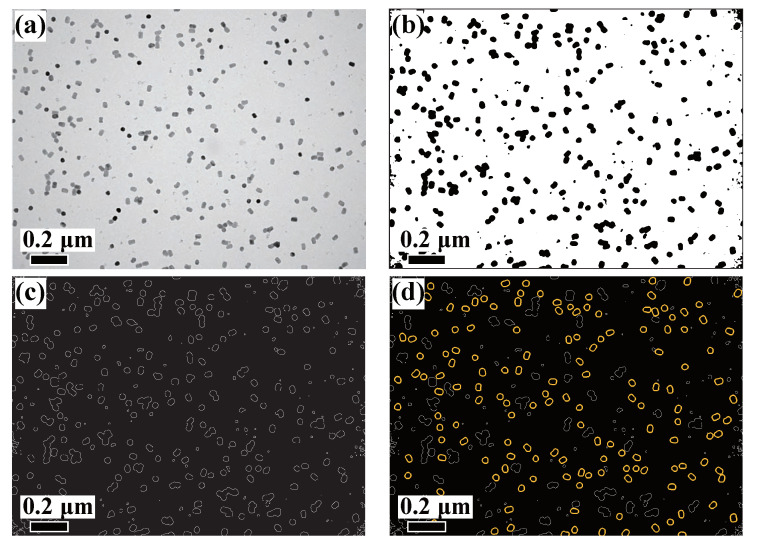
Image pre-processing of the UCNPs TEM image. (**a**) A low-mag, BF-TEM image for well dispersed UCNPs sample. (**b**) Binary image after the dynamic background processing. (**c**) Particle edges detected by the Canny Edge detection method. (**d**) Convexity filtered particle contours (yellow), where all the non-convex particle aggregates (grey) are filtered out.

Figure 3a shows bright-field (BF)-TEM images of NaGdF${}_{4}$: 49%Yb, 1%Tm nanoparticles. This image represents a typical situation of a relatively low magnification TEM image showing well dispersed nanoparticles. This type of image is commonly used to evaluate the particle synthesis process, as lower magnification images have the advantage of capturing more particles in the field of view, thus providing better statistical accuracy for a given amount of experimental data and time. The trade-off is that lower magnification images ultimately imply fewer pixels for describing a given particle contour. Therefore, the deliberately chosen low magnification image is served to test the robustness of our method in capturing the particle shapes.

**Figure 4:**
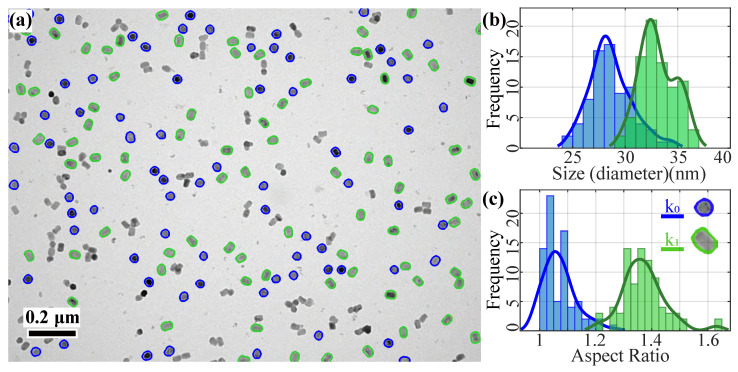
Shape classification results for the TEM image of UCNPs. (**a**) The two different shape classification particle contours are marked with two colors. (**b**) Size distribution (approximate diameter assuming the particles are spherical) of the two clusters, k${}_{0}$ and k${}_{1}$. (**c**) Aspect ratio distribution of the two clusters.

Figure 3b–d shows the successful processing by our pre-processing method, where 156 isolated particle shape contours were extracted. The optimal number of clusters, k, is determined to be two by the CVIs. The classified particles overlaid with the original TEM image are shown in Figure 4a. It can be seen that one particle shape cluster, k${}_{0}$ (blue), is polygonal (hexagons and pentagons) and the other cluster, k${}_{1}$ (green), is rod-shaped. The particle size distribution and the aspect ratio distribution for each cluster are plotted in Figure 4b,c, respectively. The two shape clusters are clearly separated in the aspect ratio distribution plot, with the polygon cluster, k${}_{0}$, having a mean value close to 1 and a higher value of about 1.4 for k${}_{1}$. In addition, the particles in the k${}_{0}$ cluster mostly have a smaller size with a mean diameter around 34 nm whereas those in the k${}_{1}$ cluster have a slightly larger mean diameter around 38 nm. Table 1 provides a summary of the statistics for the UNCP size and shape analysis.

These results demonstrate the accuracy of our method in particle contour determination and shape classification, even at relatively low image magnification.

In this case, we also implemented manual particle shape analysis as a validation of our method. For the same selected convex particles, the manual analysis results are 76 (polygonal) vs. 80 (rod-shaped), with an agreement rate of 98.7%.

### 3.2. High Packing Density Semiconductor Quantum Dots

Semiconductor quantum dots (QDs) are nanocrystals typically in the size ranges of 1–10 nm, exhibiting quantum confinement effects in their optical and electronic properties [70]. Such properties give semiconductor QDs great promise in applications such as bio-medicine [71], and light-emitting diodes [72]. The CdS/ZnSe used in this case are typical core-shell structure semiconductor QDs, with a shell layer that gives the particles better photoluminescence quantum yields and optical stability compared to bare cores [73]. It has been shown that by controlling the size of such QDs [70,74], modulation of their band gap and optical properties can be achieved. In addition, the shape of the QD particles reflects the formation of its shell layer [26]. This highlights the importance of precise control over their size and shape in their synthesis. Here we intentionally selected a batch of the CdS/ZnSe sample particles whose size was controlled but with non-distinct particle shapes.

**Figure 5:**
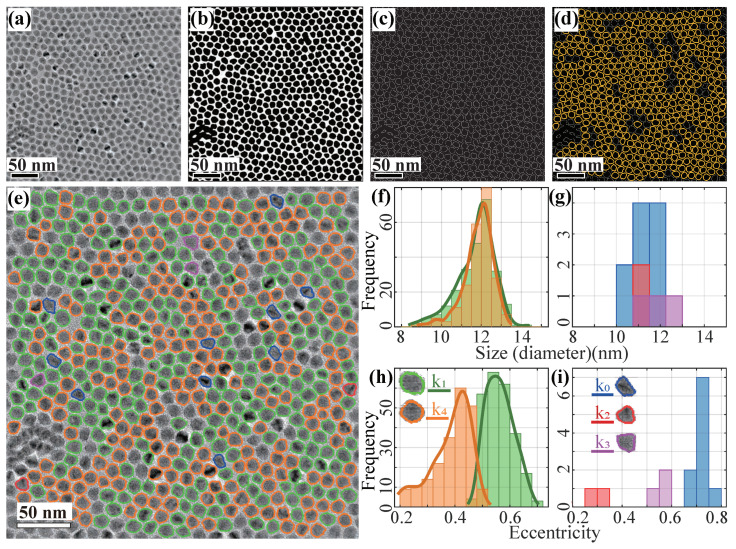
Image pre-processing and shape classification of the QDs. (**a**) BF-TEM image of the CdS/ZnSe QDs sample. (**b**) Binary image after the dynamic background processing. (**c**) Particle edges detected by the Canny Edge detection method. (**d**) Convexity filtered particle contours (yellow), where all the non-convex particle aggregates (grey) are filtered out. (**e**) Classified particle contours labeled with different colors overlaid with the BF-TEM image shown in (a). (**f**) Size distribution of the two highest population clusters. (**g**) Size distribution of the three lowest population clusters. (**h**) Eccentricity distribution of the high population clusters of k${}_{1}$ and k${}_{4}$. (**i**) Eccentricity distribution of the low population clusters.

As seen from the original BF-TEM image in Figure 5a, the QDs have a very high packing density, filling nearly the entire image where the background only occupies a very small proportion of the image. In addition, the QD contrast is relatively low compared to the background. Such complex background condition presents a challenge for background removal. Moreover, most of the particles have non-distinct shapes, making manual classification of the shape nearly impossible. Therefore, this case is deliberately selected and served to test the reliability of our method for images with low contrast, low background area and irregular particle shapes.

Figure 5b–d demonstrates the effectiveness of our pre-processing method even when little background area is present. A total of 482 isolated QDs in the image (Figure 5d, yellow) were successfully extracted for classification.

Our method determines the optimal number of clusters, k${}_{n}$ = 5. The classified particles overlaid with the original image are shown in Figure 5e. The corresponding size distribution (Figure 5f,g for high and low population clusters, respectively) and eccentricity distributions (Figure 5h,i for high and low population clusters, respectively) are plotted. Table 2 summarizes the detailed QD size and shape analysis. We can see that, as expected, all shape clusters have very similar average sizes, ranging from 11 to 12 nm.

The two shape clusters, k${}_{1}$ (green) and k${}_{4}$ (orange), with the highest population fraction (51.5% and 45.4%) have almost identical size distributions. Their aspect ratio distributions (see Supplementary Information Figure S2) also have a large degree of overlap with the k${}_{4}$ (orange) shape cluster, which has a narrower aspect ratio distribution, close to 1, whereas the k${}_{1}$ (green) shape cluster has a more dispersed aspect ratio distribution ranging from 1 to 1.4, peaking at 1.15. The apparent differences between these two shape clusters are in their eccentricity distributions (Figure 5h) with the former peaked at 0.38 and the latter at 0.56.

The other three lower population shape clusters also exhibit their own characteristics, such as the significantly larger aspect ratio of cluster k${}_{0}$ (blue), the triangular shape of cluster k${}_{2}$ (red) with narrow size distribution, and the fan shape of cluster k${}_{3}$ (purple). Their eccentricity distributions (Figure 5i) also exhibit clear differences from each other.

Such subtle differences in particle shapes would have been difficult to distinguish by human manual classification, but our ML algorithm demonstrates its effectiveness in this case.

### 3.3. Iron Nanocubes from ADF-STEM Images

Iron nanocubes combine advantages of small particle size and large magnetization, which make them an ideal tool for bio-imaging, especially magnetic particle imaging [75,76]. In the synthesis of such magnetic nanoparticles, both their shape and size can significantly affect their magnetic properties [49,77]. Therefore, the accurate identification and statistical analysis of its morphological information is crucial to realize its potential for bio-medical and thermal therapy applications. In this case study, the shapes the Fe-core/Fe${}_{2}$O${}_{3}$-shell nanoparticles are engineered to be cube-shaped to optimize their magnetic properties for biomedical imaging applications.

In this section, we use images acquired in ADF-STEM mode to demonstrate the versatility of our method in different TEM modalities. Due to the high sensitivity of the ADF-STEM mode in terms of the mass–thickness contrast, as well as the high spatial resolution it can achieve, it is often used for nanoparticles with higher atomic number compositions, as well as for high-resolution particle structure imaging. Compared to BF-TEM, ADF-STEM images generally have weaker background noise, which reduces the difficulty of background pre-processing. In addition, the contrast of ADF images provide local composition and/or particle thickness information. However, ADF-STEM imaging mode has some limitations, as it often takes longer image acquisition time than TEM mode and generally has a smaller field of view (FoV).

**Figure 6:**
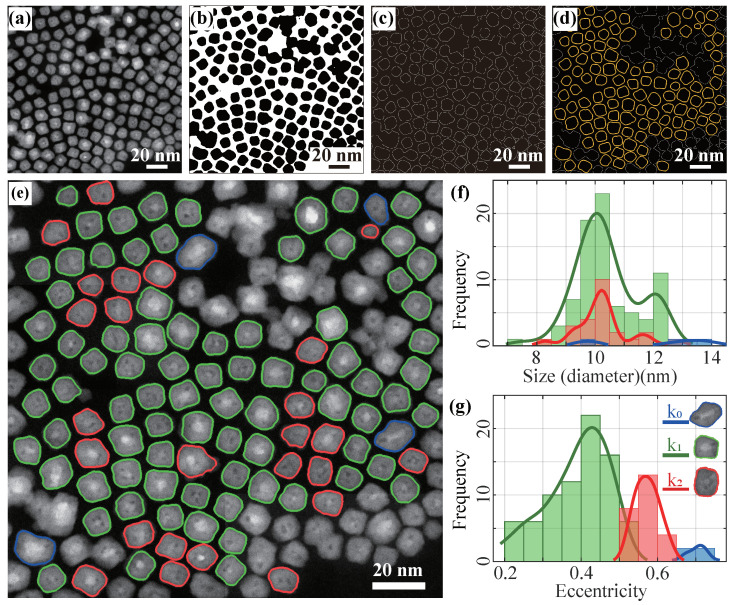
Image pre-processing of the iron cubes ADF-STEM image. (**a**) A high magnification, closely packed ADF-STEM image for the core-shell iron nanocubes sample. (**b**) Binary image after the dynamic background processing. (**c**) Particle edges detected by the Canny Edge detection method. (**d**) Non-convex particle contours (yellow) data collected as a dataset. (**e**) The three different shape classification particle contours are marked with three colors. (**f**) Size distribution (approximate diameter assuming the particles are spherical) of the three clusters. (**g**) Eccentricity distribution of the three clusters.

The ADF-STEM image of the iron nanocubes (Figure 6a) shows that the background is more homogeneous and particle-background contrast is sharper (compared to BF-TEM images). The iron nanocubes in the image also have a high packing density, with some of the particles touching each other forming aggregates. Although the iron nanocubes generally reflect their cubic shape, it is clear that there are variations in the shapes of the particles, which are also difficult to classify manually.

Again, our image pre-processing method works well for the ADF-STEM images, with 110 contours of iron nanocubes extracted (Figure 6d). Our method automatically classifies the shape of the particles into three clusters, where k${}_{0}$ (blue) represents elongated shapes, k${}_{1}$ (green) regular cube shapes and k${}_{2}$ (red) slightly distorted cube shapes, as shown in Figure 6e. Although the shape clusters do not reflect a clear distinction from their particle size distributions (Figure 6f), and the difference in their aspect ratio distributions is subtle (see Supplementary Information Figure S3), their apparent shape differences can be found in their eccentricity (Figure 6g and Table 3), where the eccentricities of the three shape clusters are concentrated at 0.4, 0.6 and 0.7, respectively.

Particles in the two highest population shape clusters accounted for over 96% of all particles, indicating that overall, the particles were close to cubic-shaped in the sample synthesis. On the other hand, the lowest population k${}_{0}$ (blue) cluster has an aspect ratio around 1.3 (Table 3), a larger eccentricity (Figure 6g) and larger particle size (Figure 6f), making it easily distinguishable from the other two clusters. This cluster represents the few particles that did not form a cubic shape in the synthesis.

## 4. Conclusions

In this work, we have demonstrated an efficient, robust and highly automated, unsupervised ML method for analyzing the nanoparticle size and shape distributions from TEM images. Our method offers several advantages over the existing methods: Firstly, it utilizes the dynamic thresholding method that can effectively remove inhomogeneous background even with low nanoparticles contrast. Secondly, it uses a hierarchical clustering method for particle shape classification, which does not require any potentially biased, user pre-determined numbers of particle shape clusters. Lastly, the use of the Cluster Validation Indexes (CVIs) contributes to determining the optimum number of particle shape clusters and offers a fully-automated analysis method without any a priori input.

The effectiveness of our method is demonstrated on three distinct examples that cover the scenarios of different particle shapes, image magnification, image background qualities, and imaging modalities. Our results show that the unsupervised ML method that we have developed provides highly automated and accurate analysis of size and shape distribution over large numbers of nanoparticles. Moreover, even for irregularly shaped particles, our method is sufficiently accurate and robust to extract and correctly classify their different shape features.

The ability to statistically analyze the nanoparticle key performance parameters of the size and shape from TEM images will certainly have a significant contribution in advancing the control of nanoparticle synthesis, thereby giving better control of particle performance. Moreover, while our methodology was only demonstrated on TEM images, it should be directly applicable to other types of microscopy images and therefore provide a broader impact to other fields of microscopy.

## Figures and Tables

**Table 1:** Clustering summary for UCNP shape and size analysis. k${}_{n}$ is the cluster number, ∑ and rate are the number and the fraction of particles for each cluster. Size and aspect ratio represents the mean size (diameter) and the mean aspect ratio of the particles for each cluster.

k${}_{n}$	∑	Rate	Size (nm)	Aspect Ratio
k${}_{0}$	75	48.1%	33.6 ± 2.0	1.07 ± 0.06
k${}_{1}$	81	51.9%	38.1 ± 1.7	1.37 ± 0.08

**Table 2:** Clustering summary for the QD size and shape analysis. k${}_{n}$ is the cluster number, ∑ and rate are the number and the fraction of particles for each cluster. Size, aspect ratio and eccentricity represent the mean size (diameter), the mean aspect ratio and the mean eccentricity of the particles for each cluster.

k${}_{n}$	∑	Rate	Size (nm)	Aspect Ratio	Eccentricity
k${}_{0}$	10	2.1%	11.2 ± 0.6	1.34 ± 0.11	0.71 ± 0.02
k${}_{1}$	248	51.5%	11.8 ± 0.9	1.14 ± 0.08	0.56 ± 0.05
k${}_{2}$	2	0.4%	11.5 ± 0.0	1.05 ± 0.03	0.29 ± 0.04
k${}_{3}$	3	0.6%	11.7 ± 0.7	1.20 ± 0.11	0.57 ± 0.03
k${}_{4}$	219	45.4%	11.9 ± 0.7	1.06 ± 0.04	0.38 ± 0.08

**Table 3:** Clustering summary for the iron nanocubes size and shape analysis. k${}_{n}$ is the cluster number, ∑ and rate are the number and the fraction of particles for each cluster. Size, aspect ratio and eccentricity represent the mean size (diameter), the mean aspect ratio and the mean eccentricity of the particles for each cluster.

k${}_{n}$	∑	Rate	Size (nm)	Aspect Ratio	Eccentricity
k${}_{0}$	4	3.6%	12.3 ± 1.6	1.33 ± 0.07	0.70 ± 0.02
k${}_{1}$	81	73.6%	10.4 ± 1.1	1.06 ± 0.04	0.39 ± 0.09
k${}_{2}$	25	22.7%	10.1 ± 1.3	1.15 ± 0.08	0.57 ± 0.03

## Data Availability

The data are available from the corresponding author upon reasonable request.

## Supplementary Materials

The following are available at https://www.mdpi.com/article/10.3390/nano11102706/s1, Figure S1: Divisive and agglomerative Hierarchical clustering representation, Figure S2: Shape classification result of High packing density QDs, Figure S3: Shape classification result of Iron nanocubes ADF-STEM images.
