# Supplementary material for: Statistically Representative Metrology of Nanoparticles via Unsupervised Machine Learning of TEM Images
Source: Nanomaterials (Basel). 2021 Oct 14;11(10):2706. doi: 10.3390/nano11102706 (PMC8539342; doi:10.3390/nano11102706)
Supplement: Supplementary file 1 [file nanomaterials-11-02706-s001.zip › nanomaterials-1341036-SI.pdf]

Supporting Information for "Statistically representative metrology of nanoparticles via unsupervised machine learning of TEM images"

Haotian Wen

School of Materials Science and Engineering,

University of New South Wales, Sydney, Australia

José María Luna-Romera and José C. Riquelme

Software and Computing Systems, Universidad de Sevilla, Seville

Christian Dwyer

Electron Imaging and Spectroscopy Tools,

PO Box 506, Sans Souci, NSW 2219, Australia

Shery L.Y. Chang

Electron Microscope Unit, Mark Wainwright Analytical Centre,

University of New South Wales, Sydney, Australia and

School of Materials Science and Engineering,

University of New South Wales, Sydney, Australia

I. SHAPE CLASSIFICATION ALGORITHMS

In this section, we explain in detail what algorithms are used to classify the nanoparticle shape information that is parameterized.

A. Clustering Method

In order to develop a general-purpose, highly automated particle shape classification method, commonly used methods for unsupervised machine learning like K-means[1] that requires a predetermined optimal number of clusters are not suitable. Therefore, we need a clustering method that does not require a pre-determined, optimum number of clusters. Hierarchical clustering algorithm is a type of unsupervised machine learning algorithm that meets such a requirement. Briefly speaking, a hierarchical clustering algorithm groups together data points (in our case, the particle contours) with similarities between them. This is achieved by using a measurement metric (in our case, the ”distance” between the pair of data points) and a linkage criteria which specifies the similarity of data sets as a function of pairwise distances of observations in the sets[2].

There exist two types of hierarchical clustering algorithms which are represented in Figure S1:

- Divisive or descending: where the clustering starts with a single cluster which includes all the elements and the process divides it successively by forming smaller clusters until there are as many clusters as elements in the dataset.
- Agglomerative or ascending: this type of hierarchical clustering is just the opposite of the descending one. It starts with as many clusters as elements in our dataset, and at each step of the algorithm, it joins those two elements that are the nearest, by this way it creates a new cluster formed by these points, and repeat the process until it ends with a single cluster made up of all available data.

In this work we have applied the agglomerative clustering method for our particle shape data. By using this method, we can automatically iterate through all possible numbers of particle shape clusters from the maximum to 1. Use these possibilities of clustering, we can then apply the CVIs (details see Section I C) to determine of the optimal number of clusters.

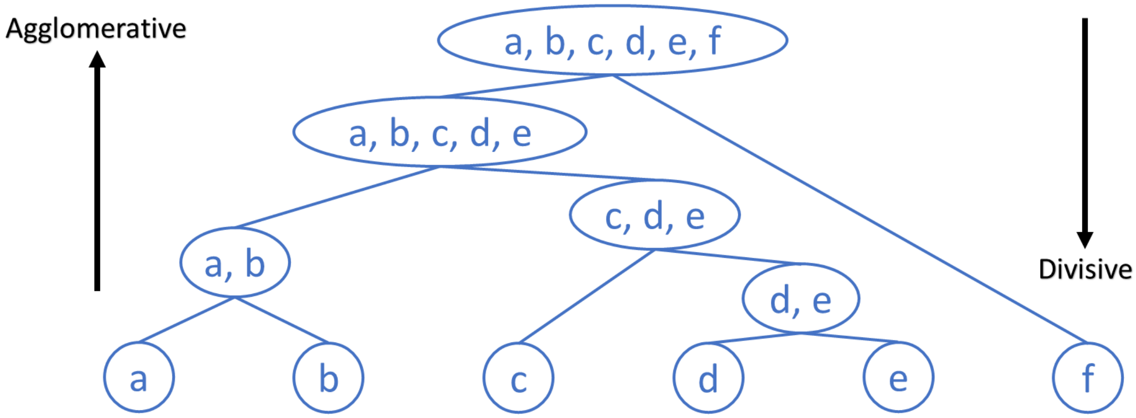

Figure S1. Divisive and agglomerative Hierarchical clustering representation

B. Average Linkage to measure the difference

In applying the agglomerative clustering method to our parameterized nanoparticle shape data, we used the Average Linkage to measure the distance, or dissimilarity, between different clusters. Average Linkage is a commonly used function in agglomerative clustering [3], which measures the distance between two clusters by calculating the average distance between all the elements in a given cluster, i, and all the elements in another cluster, j. It is a more balanced distance measurement method compared to other Linkage criteria.

C. Cluster Validity Indexes to determine the optimum number of clusters

In order to determine the optimum numbers of clusters, we discuss in this section the automatic approach by applying internal Cluster Validity Indexes (CVIs). The three internal CVIs we chose are Silhouette[4], Davies-Bouldin[5], and Calinski-Harabaz [6], and the definition of each CVI is given below. The optimum number of clusters are the common solution given by the three CVIs. The use of 3 CVIs instead of one avoids bias towards a given set of a validation criteria.

Let the dataset defined by D, and n the number of objects in D, c the center of D, and NC the number of clusters. C_i the i-th cluster, n_i the number of objects in C_i, c_i the center of C_i, and $d(x, y)$ the distance between x and y.

Let Ω be the space of the objects with a given distance d.

Then $\{A_k\}_{k=1..N}$ is a set of clusters so that $\bigcup_k A_k = \Omega$, and $A_i \cap A_j = \emptyset \quad \forall i \neq j$.

C_k is the centroid of A_k, and C_0 the centroid of Ω.

- **Silhouette**[4] performs the validation of the clustering solution based on the the pair-wise difference of inter-cluster and intra-cluster distances. The values of Silhouette are in the range $[-1, 1]$, where the best clustering solution are given when it is maximised, and is defined by Eq 1 as:

$$S = \frac{1}{NC} \sum_{i} \left\{ \frac{1}{N_i} \sum_{x \in C_i} \frac{b(x) - a(x)}{max|b(x), a(x)|} \right\} \tag{1}$$

where $b(x)$ and $a(x)$ define the inter-cluster and intra-cluster distances respectively.

- **Davies-Bouldin** [5] assesses how distant clusters can be in order to make them higher quality. Therefore, we will choose the first minimum of the Davies-Bouldin value chart to create a better model. The index is defined as follows:

$$Davies\text{-}Bouldin = \frac{1}{N} \sum_{i}^{N} \sum_{j}^{N} \max_{i \neq j} \frac{r_i + r_j}{d(C_i, C_j)} \tag{2}$$

where r_i and r_j are represented in Eq. 3, and $d(C_i, C_j)$ is the distance between the centroids C_i and C_j.

$$r_k = \frac{1}{|A_k|} \sum_{x_i \in A_k} d(x_i, C_k) \tag{3}$$

- **Calinski-Harabasz**(CH) [6] evaluates the clustering solution based on the average between- and within-cluster sum of squares. This index is given by:

$$CH = \frac{\sum_i n_i d^2(c_i, c)/(NC - 1)}{\sum_i \sum_{c \in C_i} d^2(x, c_i)/(n - NC)} \tag{4}$$

As the previous CVI's, CH also has to be maximised to find the optimal number of clusters.

It should be noted that each CVI may give different solutions since they may have more than one local maxima on each CVI, so that, each CVI will show different solutions and they may not match between each other. This is largely depending on the quality of the data. If the data quality is poor and CVIs don't have common solutions, then, Majority

Voting Methodology (MVM) needs to be applied [7]. MVM combines the results obtained of the application of the three CVI's above as a single result. MVM establishes a voting system that sets the optimal number of clusters based on the most voted solution based on the one given by the CVI's. The voting system will evaluate the results of all the CVI's so that a final single result is defined as the optimal number of clusters to group our dataset. It should be noted that the indices that indicate the quality of the clustering once performed.

II. QUANTUM DOTS AND IRON CUBES CASE STUDIES

In this section we provide additional evaluations of our shape classifications in the case studies of quantum dots and iron cubes.

A. High packing density semiconductor quantum dots

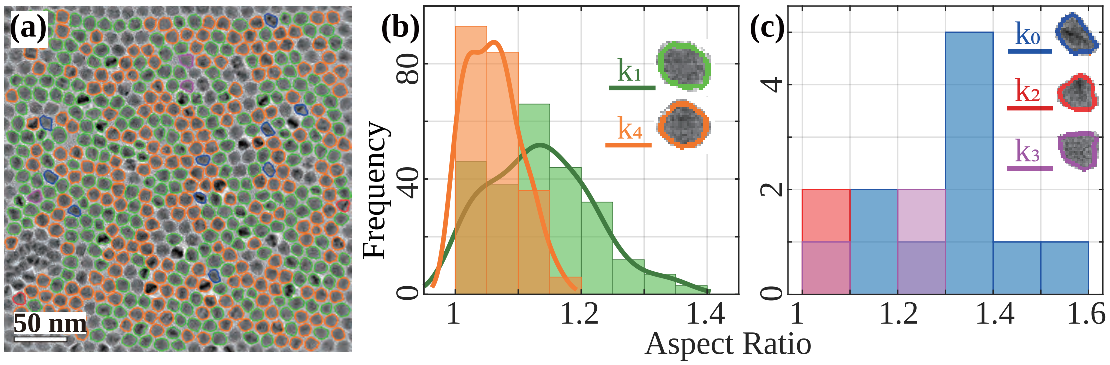

Figure S2. (a) Classified particle contours labelled with different colors overlaid with the BF-TEM image. (b) The aspect ratio distributions of the two highest population clusters, k_1 (green) and k_4 (orange). (c) The aspect ratio distributions of the other three lower population clusters.

In addition to the eccentricity distributions in the main text, the aspect ratio distributions of the QD clusters are also another shape feature attribute of interests, despite their distribution overlaps between shape clusters. For the cluster with the highest popultation, the k_4 (orange) cluster has a narrower aspect ratio distribution, close to 1, whereas the k_1 (green) cluster has a more dispersed aspect ratio distribution ranging from 1 to 1.4 peaking at 1.15. This indicates that k_4 tends to be more square or circular compared to k_1.

B. Iron nanocubes from ADF-STEM images

As shown in Figure S3, the aspect ratio distributions of the three clusters could partially show the difference in their shapes although the aspect ratio is not the main feature of difference between clusters compared to eccentricity distribution. For example, the aspect ratio of the shape cluster, k_1 (green), peaks close to 1, while the k_2 (red) cluster has a rather broad distribution peaking at a slightly higher value of 1.2, and the k_0 (blue) cluster has higher aspect ratio distribution. However, the broad distribution and overlap undermines the interpretation of the particle shape classification results by the aspect ratio distribution, compared to the more pronounced eccentricity figure distribution in the main text.

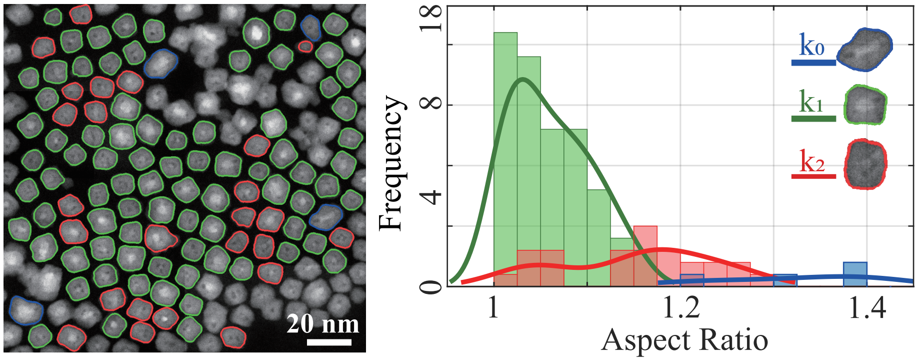

Figure S3. (Left) Classified particle contours labelled with different colors overlaid with the ADF-STEM image..(Right) The aspect ratio distributions of the three clusters.

The above distribution curves in II A and II B are normalised by a nonparametric kernel-smoothing distribution using MATLAB, such fitting curves works well with continuously distributed samples.

[1] J. MacQueen *et al.*, in *Proceedings of the fifth Berkeley symposium on mathematical statistics and probability*, Vol. 1 (Oakland, CA, USA, 1967) pp. 281–297.

[2] N. Rajalingam and K. Ranjini, International Journal of Computer Applications **19**, 42 (2011).

[3] A. K. Jain, Pattern Recognition Letters **31**, 651 (2010).

[4] P. Rousseeuw, Journal of Computational and Applied Mathematics **20** (1987), 10.1016/0377-0427(87)90125-7.

[5] D. L. Davies and D. W. Bouldin, IEEE Transactions on Pattern Analysis and Machine Intelligence **1**, 224 (1979).

[6] T. Caliński and H. JA, Communications in Statistics - Theory and Methods **3**, 1 (1974).

[7] R. Pérez-Chacón, J. M. Luna-Romera, A. Troncoso, F. Martínez-Álvarez, and J. C. Riquelme, Energies **11** (2018), 10.3390/en11030683.
